# Triple Negative Breast Cancer: Nanosolutions for a Big Challenge

**DOI:** 10.1002/advs.201500053

**Published:** 2015-07-17

**Authors:** Tânia Filipa S. Mendes, Leon D. Kluskens, Lígia Raquel Rodrigues

**Affiliations:** ^1^ Centre of Biological EngineeringUniversity of Minho4710–057BragaPortugal

**Keywords:** cancer therapy, diagnostics, drug delivery, nanotechnology, triple negative breast cancer (TNBC)

## Abstract

Triple negative breast cancer (TNBC) is a particular immunopathological subtype of breast cancer that lacks expression of estrogen and progesterone receptors (ER/PR) and amplification of the human epidermal growth factor receptor 2 (HER2) gene. Characterized by aggressive and metastatic phenotypes and high rates of relapse, TNBC is the only breast cancer subgroup still lacking effective therapeutic options, thus presenting the worst prognosis. The development of targeted therapies, as well as early diagnosis methods, is vital to ensure an adequate and timely therapeutic intervention in patients with TNBC. This review intends to discuss potentially emerging approaches for the diagnosis and treatment of TNBC patients, with a special focus on nano‐based solutions that actively target these particular tumors.

## 1 Introduction

This is an open access article under the terms of the Creative Commons Attribution License, which permits use, distribution and reproduction in any medium, provided the original work is properly cited.

It is foreseen that, for 2015, breast cancer will be one of the most commonly diagnosed cancers, accounting for about 1.7 million new cases and resulting in more than 580 thousand deaths in the US alone.^1^ This complex and heterogeneous disease is characterized by distinct cellular origins, mutations, histology, progression, metastatic potential, therapeutic response and clinical outcome.^2^ Due to its heterogeneity, additional sub‐classifications of breast cancer have been proposed based on intrinsic histological, immunopathological, and molecular features. However, only the immunopathological classification has been shown to significantly help clinicians in the therapeutic decision‐making process.^3^ The immunopathological classification of breast cancer is based on the expression of estrogen and progesterone receptors (ER/PR) and amplification of the human epidermal growth factor receptor 2 (HER2). Distinct combinations of the presence (+) or absence (–) of these receptors permit the categorization of breast tumors into four individual groups, namely ER+/PR+/HER2+; ER+/PR+/HER2–; ER–/PR–/HER2+; and ER–/PR–/HER2–, or triple negative.^4^ Absence of ER/PR has been strictly defined as less than 1% expression by the most recent American Society of Clinical Oncology/College of American Pathologists (ASCO/CAP) guidelines.^5^

HER2‐negative breast cancers (ER+/PR+/HER2– group) constitute the most prevalent immunopathological subtype, representing more than 66% of all cases, followed by triple negative breast cancers (TNBC), which occur in about 19% of the cases. The remaining events are commonly HER2‐overexpressing breast cancers either or not presenting ER/PR receptors.^4^

Currently in clinical practice, the presence of ER is considered a good indicator of overall outcome and is commonly used to identify tumors that may respond to anti‐estrogen hormonal therapy targeting ER‐dependent signaling pathways.^6^ HER2‐overexpressing tumors used to be characterized by a poor outcome, but with the advent of anti‐HER2 monoclonal antibodies targeting HER2‐dependent signaling pathways, these results have improved.^6^ Absence of PR, ER, and HER2 characterizes the TNBC subtype, which is known as the only subgroup lacking targeted therapeutic options. Moreover, this group presents the worst prognosis when compared to the other breast cancer groups due to its aggressive and metastatic nature, low response to existing therapies and high rates of relapse.^7^ Generally, TNBC is associated with women of African‐American ethnicity, with less than 40 years of age at the time of initial diagnosis and carrying BRCA1 (breast cancer 1, early onset) gene mutations.^8^, ^9^, ^10^ From a morphological point of view, approximately 90% of TNBC occurrences are invasive ductal carcinomas, whereas the remaining cases are classified as apocrine, lobular, adenoid cystic, and metaplastic.^11^ Curiously, the prognosis of each class has shown to be distinct despite sharing the triple‐negative phenotype. This heterogeneity has also been confirmed by gene expression profile analyses of breast cancer datasets. These studies led to the identification of six distinct TNBC subtypes that include basal‐like 1 (BL1), basal‐like 2 (BL2), mesenchymal‐like (ML), mesenchymal stem‐like (MSL), luminal androgen receptor (LAR) and immunomodulatory (IM).^12^ The diversity of TNBC in terms of gene expression subtypes and the repertoire of genetic events has been recently reviewed.^13^ The intrinsic complexity of TNBC, usually resulting in distinct response of patients to therapy, suggests the need for a further subdivision of this subtype from a clinical perspective. The identification of specific molecular markers for TNBC subgroups would undoubtedly contribute to a more precise diagnosis, making the development of predictive biomarkers and targeted therapies possible. Although an optimal treatment for TNBC remains an unmet need, this review aims at discussing the potentially emerging approaches for its diagnosis and treatment, with particular emphasis on nanotechnology‐based strategies.

## 2 TNBC: Strategies for Diagnosis and Treatment

Significant improvements in medical instrumentation combined with recent advances in nanotechnology and synthetic biology have contributed to the progress of the oncology field. Particularly, several nanocarriers and targeting agents are currently under investigation for delivering therapeutic and imaging agents at the tumor site towards an improvement of diagnosis and therapy.

### 2.1 Current Scenario in the Diagnosis of TNBC

The aggressive and metastatic nature of TNBC makes its diagnosis particularly important and decisive to ensure an early and adequate therapeutic intervention. In clinical practice, breast cancer diagnosis generally relies on three main types of analyses: i) clinical examination through palpation; ii) radiological exams, including mammography, ultrasonography, and magnetic resonance imaging (MRI); iii) pathological tests based on biopsies.^14^ Mammography, the most widely applied radiological exam for breast cancer detection, uses low‐energy X‐rays to create images of patients' breasts allowing the visualization of abnormal tissue features.^15^ However, clinical data indicate that most TNBC tumors lack the abnormal features of breast cancer, leading to an inaccurate diagnosis.^15^, ^16^ Complementary exams, such as ultrasonography, should therefore be considered when evaluating patients with increased risk of TNBC. Ultrasonography allows the visualization of internal body structures through ultrasound images and typically presents a sensitivity higher than 90% for TNBC detection.^17^ However, its accuracy is greatly dependent on the examiner's experience and may be limited in case of tumors presenting benign image features.^15^, ^16^, ^17^ MRI uses magnetic fields and radio waves to construct images of the body and has been more accurate in TNBC diagnosis.^15^, ^16^ Despite the essential role of radiological examination of suspicious breast cancer patients, it frequently results in false‐positive findings leading to unnecessary invasive biopsy analyses.^15^ On the other hand, many early stage tumors remain unnoticed until they progress and first symptoms, such as breast pain and nipple discharge appear.^15^ Therefore, clinical identification of TNBC currently relies on determining the absence of ER, PR, and HER2 in biopsy samples using standard immunohistochemistry (IHC) tests.^14^ IHC analyses enable the detection of those cell receptors through the use of antibodies that specifically bind to antigens present in the tissue samples.^14^ Antibody–antigen binding is commonly visualized using chemical or enzymatic staining.^14^ Several standards and recommendations for IHC analyses have been proposed by international experts to improve reproducibility and reliability of results amongst laboratories.^5^, ^18^ In summary, IHC tests and critical examination by clinicians are the best available approaches to validate a TNBC diagnosis.

### 2.2 Novel Approaches for the Diagnosis of TNBC

Novel or optimized methods and biomarkers that provide unequivocal information about TNBC at early stages, as well as predictive indications about the therapeutic outcome have been pursued by both clinicians and researchers. Recent studies suggested that positron emission tomography (PET) with 2‐deoxy‐2‐[fluorine‐18]fluoro‐d‐glucose (^18^F‐FDG) could be a promising tool for the detection of TNBC and axillary lymph node metastasis with a higher level of accuracy compared to other tumor subtypes.^15^ PET is an imaging method that constructs three‐dimensional images by measuring radiolabelled tracer molecules in the body.^15^ ^18^F‐FDG tracer, a glucose analogue, is taken up by cells and allows the identification of regions with increased glucose uptake—a characteristic of tumor tissues.^15^ This technique enables the detection of metabolic alterations even before morphological changes take place.^19^ However, the use of ^18^F‐FDG–PET technique as a diagnostic tool for TNBC may be limited to metastatic stages due to the low FDG uptake in early breast cancers.^15^ Although anatomic imaging techniques usually apply contrast agents (e.g. microbubbles or radioactive, fluorescent, and bioluminescent probes) to improve the resolution of images, most are nonspecific, exclusively providing morphologic information or allowing tumor detection only in advanced stages.^20^

**Figure 1:**
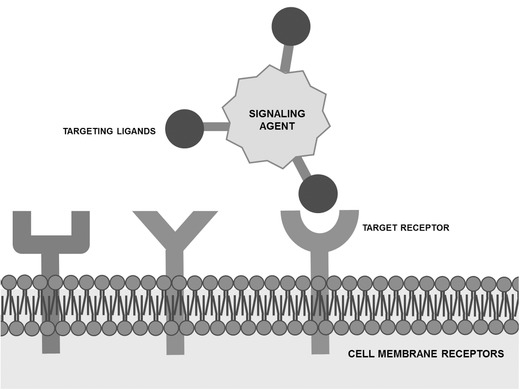
Schematic representation of a molecular probe containing a signaling agent conjugated to targeting ligands that are able to recognize specific cancer cell receptors.

Advances in the synthetic biology field have contributed to develop novel and specific contrast agents with potential molecular imaging applications.^21^, ^22^ Briefly, molecular imaging uses molecular probes to detect key biological processes by coupling signaling or contrast agents with ligands that target overexpressed or upregulated cellular receptors (**Figure** **1**). This approach allows the examination of tumors from a molecular rather than a morphological perspective.^23^ Since molecular changes generally occur much earlier than morphological alterations, molecular imaging may be very promising for the detection of early stage tumors. However, from our perspective, the choice of the adequate targeting ligand is crucial for the success of the detection technique.

#### 2.2.1 Targeting Ligands

Antibodies, peptides, aptamers and other small molecules have been proposed as targeting ligands to be further combined with contrast agents used in imaging techniques. Their main features, as well as examples of their potential in the detection of TNBC are discussed in this section.

*Antibodies*: Antibodies comprise the most studied class of targeting ligands. This class is characterized by single protein molecules containing two epitope binding sites that provide high selectivity and affinity for target binding.^24^ Advances in protein engineering have allowed the production of antibodies with desired features (e.g. size, specificity, immunogenicity), depending on their final use.^25^ However, the relatively high production costs of antibodies, alongside with their problematic conjugation to signaling agents, often restrict their application.^24^ Various antibodies have been proposed as suitable targeting ligands for conjugation with breast cancer molecular imaging probes. For instance, radiolabeled transtuzumab and pertuzumab antibodies targeting the HER2 cellular receptor entered clinical trials as imaging probes for PET and single photon emission computed tomography (SPECT) imaging modalities.^26^ Moreover, anti‐epidermal growth factor receptor (anti‐EGFR) and anti‐vascular endothelial growth factor receptor (anti‐VEGFR) antibodies conjugated with fluorescent nanoparticles and ultrasound contrast agents, respectively, have been assessed.^27^, ^28^ Using fluorescence microscopy imaging and ultrasonography, these antibody‐conjugated imaging agents showed the ability to effectively target breast cancer cells, enhancing the specificity and sensitivity of the imaging techniques.^27^, ^28^ A few studies have demonstrated the utility of antibodies as targeting ligands for promising targets in TNBC models. Human antibodies specifically targeting the urokinase plasminogen activator receptor (uPAR), a target present in TNBC cells, demonstrated specific probe localization to the tumors using both optical and SPECT imaging after labeled with near‐infrared (NIR) fluorophores and Indium‐111 (^111^In), respectively.^29^ Moreover, uPAR‐targeted antibodies labeled with ^111^In and Technetium‐99m (^99m^Tc) showed improved detection sensitivity for bone and soft‐tissue metastases compared to ^18^F‐FDG using PET imaging.^29^ Shi et al. have also suggested the use of a tissue factor (TF)‐targeting antibody labeled with Copper‐64 (^64^Cu) in PET imaging after in vitro validation of fast tumor uptake in a TNBC model.^30^ Although TF expression is upregulated in many solid tumor types besides TNBC, which makes this protein an interesting target from a clinical perspective, further studies need to be performed before this translation takes place.^30^ The potential of antibodies as targeting ligands transcends tumor detection purposes. Radiolabeled antibodies could be extremely useful for both radioimmunotherapy (RIT) and tumor monitoring during therapy.^31^, ^32^, ^33^ To evaluate the potential of the anti‐human B‐B4 monoclonal antibody to target the syndecan‐1 (CD138) antigen—correlated with poor prognosis and aggressive phenotypes in breast carcinoma—a preclinical study on TNBC xenograft mice was performed.^31^ Iodine‐124 (^124^I) and ‐131(^131^I) radiolabeled B‐B4 antibodies were tested for immuno‐PET imaging and RIT, respectively. Immuno‐PET using ^124^I‐B‐B4 allowed the visualization of CD138‐expressing tumors. Mice treated with ^131^I‐B‐B4 RIT experienced partial or complete responses to therapy. These results reinforced the relevance of the B‐B4 antibody for the diagnosis and treatment of metastatic TNBC. Scheltinga et al. have monitored downregulation of the insulin‐like growth factor receptor‐1 (IGF‐1R) and VEGF expression in vivo in response to heat shock protein 90 (Hsp90) inhibition therapy using Zirconium‐89 (^89^Zr)‐labeled MAB391 and bevacizumab for PET imaging.^32^, ^33^ These studies not only demonstrated the utility of ^89^Zr‐labeled antibodies in the visualization of IGF‐1R and VEGF levels in vivo, but also showed their potential as biomarkers for drugs targeting IGF‐1R and VEGF expression.

*Peptides*: Peptide‐based targeting agents are a promising class of low molecular weight ligands. They present high specificity and affinity for target binding, low immunogenicity and reduced production costs.^34^ In contrast to antibodies, peptides can target intracellular molecules. However, due to their small size, their biodistribution may be negatively affected when attached to signaling agents.^20^

The screening of Phage Display libraries has been widely used to discover target‐binding peptides.^24^ Phage Display technology consists in expressing peptide sequences fused to bacteriophage coat proteins through genetic engineering.^35^ By inserting randomized DNA sequences into the bacteriophage genome, a variety of peptides (library) can be screened over several rounds against the desired target (e.g. peptides, proteins or DNA sequences) using binding assays to identify target‐binding peptides.^24^, ^35^ Recently, a breast cancer‐targeting peptide was discovered using this technology.^36^ The identified peptide, Cys–Leu–Lys–Ala–Asp–Lys–Ala–Lys–Cys (CK3), contains a C‐end rule motif that is thought to mediate binding to the neuropilin‐1 (NRP‐1), a transmembrane protein that is overexpressed in diverse breast tumors and is usually associated with a poor outcome. The potential of the CK3 peptide as targeting ligand was validated by SPECT and near‐infrared fluorescence (NIRF) imaging techniques, which showed its accumulation in TNBC mice models. Crisp et al. rationally designed a distinct tumor imaging strategy based on activable cell‐penetrating peptides (ACPPs) targeting the matrix metalloproteinase (MMP)‐2 enzyme.^37^ This approach takes advantage of the intensified extracellular protease activity occurring in most invasive types of cancer. To amplify the specificity and sensitivity of this strategy, the cyclic‐RGD peptide was covalently linked to ACPP. This dual‐targeted mechanism resulted in enhanced tumor uptake and contrast during fluorescence imaging assays performed in in vivo models of TNBC.^37^ Additional characteristics of the extracellular tumor environment, such as its acidic pH (6.2–7.0), have been exploited for specific targeting of imaging agents using peptides. Recently, a pH‐responsive MRI nanoprobe was synthesized using a pH low insertion peptide (pHLIP) known to target tumor acidic pH.^38^ As low pH is not observed in normal tissues, pHLIP‐conjugated MRI nanoparticles were specifically internalized by TNBC cells in vitro at pH 6.5. Concomitantly, systemic delivery of the nanoparticles in a TNBC mouse model led to their accumulation in the tumor tissue, thus allowing its MRI detection.^38^

*Aptamers*: Aptamers are short, single‐stranded DNA or RNA oligonucleotides with a unique 3D conformation that bind to specific target molecules with high affinity.^39^, ^40^ This class of targeting ligands presents several advantageous features over antibodies, including low immunogenicity, high stability, easy production and modification.^40^ Target‐binding aptamers can be selected through a screening process similar to Phage Display named Systematic Evolution of Ligands by Exponential Enrichment (SELEX).^24^, ^39^ Aptamers displaying high affinity and specificity for desired target molecules (e.g. nucleic acids, proteins, sugars, and phospholipids) can be isolated from large libraries of randomized oligonucleotides over several rounds of selection.^39^, ^40^ Limitations include their susceptibility to degradation by nucleases and fast clearance. Nonetheless, these short oligonucleotides have gained increasing attention for the development of molecular probes. Their potential in targeting ligands for molecular imaging has been recently reviewed.^41^ DNA aptamers specifically targeting triple negative metastatic tumor cells have been identified using cell‐SELEX methodology.^40^ Preliminary imaging studies using a selected aptamer (LXL‐1) revealed a 76% detection rate against metastatic breast cancer tissue and suggested that a cell surface membrane protein was the target.^40^ The potential of a platelet‐derived growth factor (PDGF)‐binding aptamer conjugated to gold nanoparticles in the detection of PDGF overexpressing TNBC breast cancer cell lines has also been demonstrated.^42^ The use of this aptamer as targeting ligand led to aggregation of gold nanoparticles in the cytoplasm of cancer cells, allowing the differentiation between cancer and normal cells using a dark field optical microscope after photo‐illumination.^42^ Despite the rapidly growing interest in aptamers, with a few candidates already in preclinical and clinical trials for use as drugs, this class of molecules still needs to mature before its clinical translation as targeting ligands for molecular probes.^43^

*Small molecules*: Small molecule‐based ligands (<500 Da) are an attractive class of targeting agents due to their vast diversity in structure and properties, and relative low production costs.^21^, ^24^ However, only a few small molecules have been described as potential targeting agents for molecular imaging of tumors. The ^18^F‐FDG glucose analogue is the most widely used small molecule with clinical application in cancer imaging.^21^ Riboflavin and folate are examples of small molecules currently under investigation.^24^ Targeted ligands based on riboflavin can use the riboflavin carrier protein (RCP), which is known to be upregulated in metabolically active cells, to target metabolically active cancer or endothelial cells.^24^ Similarly, folate‐based ligands can be used to direct imaging agents to tumor cells taking advantage of the folate affinity for its receptors, which are known to be overexpressed in most tumors.^24^ The potential of folate molecules to drive an ultra‐small super‐paramagnetic iron oxide contrast agent (P1133) to cell folate receptors expressed in TNBC cells has been demonstrated both in vitro and in vivo.^44^ P1133 particles were specifically internalized by folate receptor‐expressing tumor cells, enhancing magnetic resonance images.^44^

#### 2.2.2 Image‐guided therapy

Current primary goals of novel imaging strategies include the reduction of patient's exposure to radiation and the improvement of exams resolution and specificity. However, imaging agents may also play an important role in the development of image‐guided therapies as alternative to surgery. Photothermal, shortwave radiofrequency and alternating magnetic fields ablation techniques are examples of such therapies.^45^ In all approaches, nanoparticles are activated by externally applied stimuli, namely NIR light, shortwave radiofrequency energy and alternating magnetic fields.^46^, ^47^, ^48^ Gold nanoparticles, carbon nanotubes and magnetic nanoparticles are among the most commonly studied nanomaterials for image‐guided therapy. A few studies have evaluated the efficiency of photothermal ablation in TNBC cells both in vitro and in vivo using gold nanorods or nanoparticles irradiated with a NIR laser.^49^, ^50^ The results have shown a successful elimination of tumor cells by modulating the size and concentration of particles, power of the laser and irradiation period.^49^, ^50^ However, further investigations should be carried out to evaluate the toxicity and specificity of these nanoparticles. Although a lot of work needs to be done to clarify the clinical relevance of these strategies, multifunctional approaches combining both imaging and therapeutic properties in the same nanoparticle are gaining interest and promise to revolutionize the breast cancer theranostics field.

### 2.3 Currently Approved and Emerging Therapeutic Routes for TNBC

For many years, breast cancer patients placed their hope uniquely in cytotoxic chemotherapy. Fortunately, over the past two decades, new target‐directed approaches for breast cancer treatment have been developed and approved for clinical use (**Table** **1**). The introduction of new therapeutic strategies led to a decline in the mortality rate by about 30% and an improvement of the 5‐year overall survival rate to 90%.^52^ However, life expectancy for metastatic breast cancer patients is less optimistic, with a 5‐year overall survival rate of only 24%.^53^

**Table 1:** Authorized medicines for breast cancer in the European Union (EU) by the European Medicines Agency (EMA), and in the USA by the Food and Drug Administration (FDA)

Class	Common name	Trade name	Condition	Approval date	Regulator
Mitotic inhibitors (non‐taxanes)	Eribulin mesylate	Halaven	Metastatic BCa)Locally advanced or metastatic BCa)	11/201003/2011	FDAEMA
Mitotic inhibitors (epothilones)	Ixabepilone	Ixempra	Locally advanced or metastatic BCa)	10/2007	FDA
Mitotic inhibitors (taxanes)	Albumin‐bound paclitaxel	Abraxane	Locally advanced or metastatic BCa)	01/2008	EMA
	Docetaxel	Docetaxel AccordDocetaxel KabiDocetaxel MylanDocetaxel TevaDocetaxel Winthrop	BCa)BCa)BCa)BCa)BCa)	05/201205/201201/201201/201004/2007	EMAEMAEMAEMAEMA
		Taxotere	BCa)Locally advanced or metastatic BCa)	11/199505/1996	EMAFDA
Anthracyclines	Liposomal doxorubicin hydrochloride	CaelyxMyocet	Metastatic BCa)	06/199607/2000	EMAEMA
Anti‐metabolites	Capecitabine	EcansyaCapecitabine AccordCapecitabine SUNCapecitabine Teva	Locally advanced or metastatic BCa)Locally advanced or metastatic BCa)Locally advanced or metastatic BCa)Locally advanced or metastatic BCa)	04/201204/201206/201304/2012	EMAEMAEMAEMA
		Xeloda	Locally advanced or metastatic BCa)Advanced BCa)	02/200104/1998	EMAFDA
Bisphosphonates	Pamidronate disodium	Aredia	Bone metastases	08/1996	FDA
Tyrosine kinase inhibitors	Lapatinib	Tykerb	HER2+b) advanced or metastatic BCa)	03/200706/2008	FDAEMA
Serine/threonine kinase inhibitors	Everolimus	Afinitor	ER+c)/PR+d)/HER2‐b) advanced BCa)	08/200907/2012	EMAFDA
Aromatase inhibitors	Anastrozole	Arimidex	Advanced BCa)	01/1996	FDA
	Letrozole	Femara	BCa)Advanced or metastatic BCa)	07/199701/2001	FDAFDA
SERMse)	Fulvestrant	Faslodex	ER+c) metastatic BCa)ER+c)/PR+d) metastatic BCa)	10/199802/1996	FDAEMA
	Tamoxifen	Nolvadex	ER+c) metastatic BCa)	10/1996	FDA
	Toremifene	Fareston	ER+c)/PR+d) metastatic BCa)	02/1996	EMA
Monoclonal antibodies	Bevacizumab	Avastin	Metastatic BCa)	01/2005	EMA
	Pertuzumab	Perjeta	HER2+b) locally recurrent or metastatic BCa)HER2+b) metastatic BCa)	03/201306/2012	EMAFDA
	Trastuzumab	Herceptin	Early BCa)Metastatic BCa)	08/200010/1998	EMAFDA
	(Ado)‐trastuzumab emtansine (trastuzumab linked to DM1 drug)	Kadcyla	HER2+b) locally advanced or metastatic BCa)	11/201302/2013	EMAFDA

^a)^BC: breast cancer;

^b)^HER2+/HER2‐: human epidermal growth factor receptor 2 positive/negative;

^c)^ER+/–: estrogen receptor positive/negative;

^d)^PR+/–: progesterone receptor positive/negative;

^e)^SERMs: selective estrogen‐receptor response modulators.^134^, ^135^

**Figure 2:**
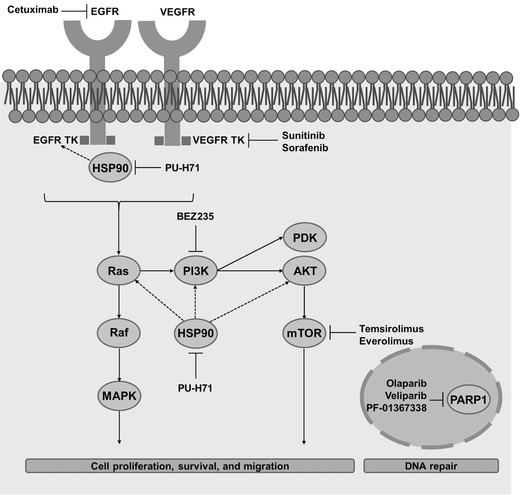
Pathways, targets and emerging targeted agents in TNBC. VEGFR: vascular endothelial growth factor receptor; EGFR: epidermal growth factor receptor; HSP90: heat‐shock protein 90; TK: tyrosine kinase; PI3K: phosphoinositide‐3‐kinase; PARP1: poly(ADP‐ribose)polymerase 1; PDK: 30‐phosphoinositide‐dependent kinase; MAPK: Mitogen‐activated protein kinase; mTOR: mammalian target of rapamycin.

Currently, the therapy selected for each patient is dictated by the presence of cellular receptors for estrogen and progesterone hormones and human epidermal growth factor 2 (HER2).^51^ However, as patients with TNBC do not present those cellular receptors, the available therapeutic routes are more limited. In addition to surgery and radiotherapy, chemotherapy remains their only option. Cytotoxic combinations of anthracyclines and taxanes are commonly administered as first‐line treatment followed by capecitabine at the time of progression.^54^, ^55^, ^56^ Anthracyclines (e.g. doxorubicin) are antitumor antibiotics that interfere with enzymes involved in DNA replication, independently of the phase of the cell cycle.^57^ Similarly, taxanes (e.g. docetaxel and paclitaxel) can damage cells in all cell cycle phases, but they act preferentially during the M phase by stopping the mitosis process or inhibiting the synthesis of proteins involved in cell reproduction.^58^ Capecitabine is a prodrug of 5‐fluorouracil (5‐FU) that belongs to the anti‐metabolites class of drugs.^59^ Generally, the 5‐FU active form inhibits the RNA synthesis and its functions, as well as thymidylate synthase activity, and is incorporated into DNA causing strand breaks and damaging cells during the S phase of the cell cycle.^59^ The limited treatment options available for TNBC together with its naturally aggressive behavior usually result in a worse prognosis compared to hormone or HER2 receptor positive tumors.^51^, ^60^ Moreover, potential resistance to anthracyclines or taxanes also limits the choices for second‐ or further‐line chemotherapy to a small number of non‐cross‐resistant regimens.^60^ Alternative chemotherapeutic agents, such as eribulin and ixabepilone—two non‐taxane mitotic inhibitors—have raised hope for patients with metastatic TNBC. Eribulin has been shown to reduce the risk of death by 29% in patients previously treated with anthracyclines or taxanes, but it is even more effective in capecitabine pretreated patients.^61^ Ixabepilone—an analogue of epothilone B—has been approved for advanced breast cancer treatment when combined with capecitabine after failure of anthracyclines and taxanes.^62^ However, there are only a few prospective randomized adjuvant trials addressing the TNBC subgroup. Most data have been retrieved from a retrospective subset analysis of larger trials including all types of non‐HER2 positive breast cancer or from neoadjuvant trials. In general, approved agents for breast cancer therapy have resulted in improvements of patient's outcome, but the prognosis for metastatic TNBC patients remains poor. Much work has been aimed at improving this scenario. Sunitinib and sorafenib—two anti‐VEGFR tyrosine kinase inhibitors—have emerged as potential candidates showing therapeutic activity in breast cancer trials with significant TNBC populations.^63^, ^64^ Cetuximab—an anti‐EGFR monoclonal antibody currently approved by the Food and Drug Administration (FDA) and the European Medical Agency (EMA) for colorectal and head and neck cancers treatment—has also shown activity in metastatic TNBC treatment when combined with cisplatin and carboplatin.^65^, ^66^ Moreover, DNA damage platinum‐based regimens are gaining particular relevance in treating BRCA mutation‐carrier patients since these genes are important regulators of DNA repair and consequent maintenance of genomic stability.^67^ Recent trials also suggested novel approaches for TNBC therapy based on agents that inhibit poly(ADP‐ribose) polymerase (PARP), mammalian target of rapamycin (mTOR), phosphatidylinositol 3‐kinase (PI3K), insulin‐like growth factor (IGF), Hsp90 and histone deacetylase (HDAC) (**Figure** **2**).^51^ However, evaluating their efficacy is usually problematic as these trials have not been specifically designed for TNBC patients.

Nanotechnology‐based drug delivery systems able to improve the therapeutic index of conventional anticancer agents have also been increasingly developed.^68^ Such efforts have led to incredible advances, with nanomedicines evolving from simple passive tumor targeting carriers to multifunctional active targeting vehicles.^69^, ^70^

#### 2.3.1 Nanocarriers

Nanomedicines for breast cancer can be applied in three distinct ways, namely through direct intratumoral delivery, passive targeting and active targeting.^45^ Intratumoral delivery of nanomedicines confines their action to the tumor site.^71^ However, as it requires direct injection guided by conventional imaging techniques, this strategy is limited to tumors that can be image‐detected.^71^ Passive targeting of nanomedicines relies on tumor‐selective enhanced permeability and retention (EPR) effect characterized by an increased accumulation of macromolecules in the tumor tissues.^72^ This phenomenon results from distinct features of the tumor microenvironment, particularly the hyperpermeability of the tumor vasculature to macromolecules and the enhanced fluid retention in the tumor interstitial space resulting from a dysfunctional lymphatic drainage system.^72^ However, the difficulty to achieve therapeutic drug concentrations at the tumor site may be a critical limitation for passive targeting.^73^, ^74^ Active targeting involves the conjugation of either monoclonal antibodies, peptides or aptamers to the surface of nanoparticles for specific binding to corresponding antigens or receptors in tumor cells.^45^, ^75^ This approach results in specific accumulation of nanomedicines at the tumor site.^45^, ^75^ In addition to minimizing systemic toxicity, nanomedicines can be tailored to offer diverse advantages over conventional therapeutic agents:

- Entrapment of poorly water soluble drugs, improving drug delivery;
- Transport of large therapeutic payloads;
- Controlled and sustained drug release, improving half‐life in blood circulation;
- Enhanced internalization into the tumor via endocytosis, avoiding recognition by the P‐glycoprotein and reducing further drug resistance;
- Combined or multimodality therapy by co‐delivery of two or more chemo‐, radio‐, thermo‐, and biotherapeutic agents, promoting or avoiding their synergistic or antagonist effects, respectively;
- Visualization of drug delivery sites by co‐delivery of therapeutic and imaging agents.^45^, ^70^, ^76^, ^77^

Despite all inherent benefits of using nanomedicines, special attention must be paid to aspects such as biodegradability, toxicity and immunogenicity of their constituents and metabolic products.^78^

Diverse formulations of nanomedicines with potential application in TNBC have been studied (**Table** **2**), with polymeric nanoparticles, polymeric micelles, dendrimers, viral nanoparticles, liposomes, carbon nanotubes and nanoconjugates as the most described in the literature. These systems can be used to deliver drugs, oligonucleotides, DNA or proteins.^79^, ^80^

**Table 2:**
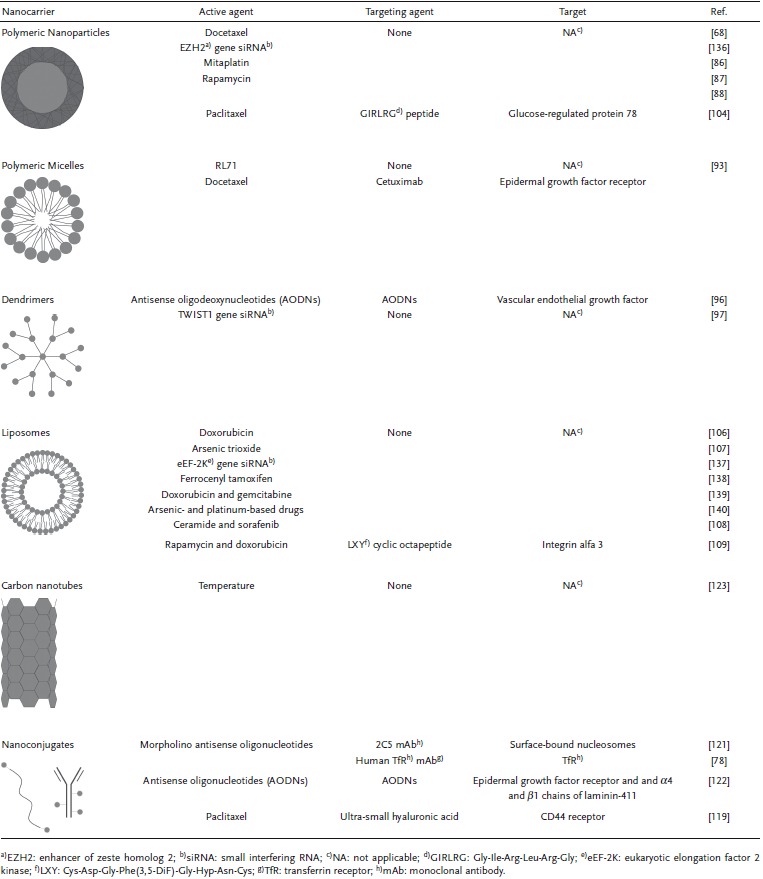
Promising nanomedicines for triple negative breast cancer (TNBC) therapy

Besides the improved effects of targeted nanomedicines, their application in TNBC treatment is limited by the lack of known highly expressed tumor targets and by the development of corresponding ligands.^78^, ^81^ Molecular biology‐based screening techniques, such as Phage Display and SELEX, may play a catalytic role in the identification of ligands that differentiate tumor cells from healthy ones, which can then be used to identify the target of interest.

*Polymeric nanoparticles*: Polymeric nanoparticles are vehicles with a diameter varying from 50 nm to over 10 μm and made of natural or synthetic polymers.^82^ Both hydrophilic and hydrophobic drugs, as well as proteins and nucleic acids, can be encapsulated into nanoparticles without chemical modification.^82^ Encapsulated agents are usually delivered at controlled rates over time or in response to the local environment.^82^ Diverse release processes, such as surface or bulk erosion, diffusion or swelling followed by diffusion are commonly observed.^69^ Polymeric nanoparticles also offer the possibility to graft, conjugate or adsorb amphiphilic polymers at their surface, improving systemic circulation half‐life.^83^, ^84^ Due to their interesting features, application of polymeric nanoparticles in cancer therapy has been extensively studied.^69^ They have been found to accumulate at a hundred times higher concentrations in tumor tissues compared to those in normal tissues, maintaining drug levels in an optimum range for longer periods of time and increasing drug efficacy.^85^ Diverse studies on specific in vitro and in vivo models of TNBC have proven nanoparticle‐encapsulated drugs (e.g. docetaxel, mitaplatin, and rapamycin) as effective as free drugs in inhibiting tumor growth even at low concentrations.^68^, ^86^, ^87^ These observations indicate the possibility to reduce therapeutic dosages, thus decreasing the side effects on healthy tissues.^68^, ^86^, ^87^ In addition to passive targeting, polymeric nanoparticles can be used to actively deliver cargoes at the tumor site when attached to specific ligands. A novel peptide (Gly–Ile–Arg–Leu–Arg–Gly) able to selectively recognize tumors expressing glucose‐regulated protein GRP78, a radiation‐induced cell surface receptor, has been identified using Phage Display.^88^ The conjugation of this peptide to polyester nanoparticles encapsulating paclitaxel induced apoptosis and delayed tumor growth in an irradiated xenograft mice model of TNBC, improving the therapeutic outcome compared to chemotherapy alone.^88^

*Polymeric micelles*: Polymeric micelles are colloidal particles with a hydrophobic core and a hydrophilic shell typically presenting 5 nm to 100 nm in diameter.^79^, ^80^ Generally, they are formed by the assembly of hydrophobic and hydrophilic polymers in aqueous environments.^79^, ^80^ The micelles' core results from Van der Waals bonds, which distribute the hydrophobic polymers symmetrically stabilized by the hydrophilic shell.^79^, ^80^ Due to their inherent amphiphilic properties, micelles can function as special vehicles for drug delivery. On one hand, the hydrophilic region makes micelles soluble in water, thus easily administrable intravenously, and avoids rapid uptake by the RES, improving circulation half‐life.^89^ On the other hand, the hydrophobic region can transport hydrophobic drugs loaded by physical encapsulation or chemical covalent attachment.^90^ The potential of polymeric micelles to deliver anticancer drugs has been suggested in various preclinical and clinical studies. NK012, a poly(ethylene glycol)‐poly(glutamic acid) (PEG‐PGlu)‐based micelle formulation carrying an irinotecan active metabolite (SN‐38), has shown significant antitumor activity with no toxicity in several orthotopic tumor models by enhancing distribution and prolonging release of SN‐38.^91^ This candidate has entered phase II trials in patients with TNBC.^91^ Styrene‐co‐maleic acid (SMA)‐based micelles have also been used to deliver RL71—a hydrophobic second generation curcumin derivative with poor bioavailability—in various TNBC cell lines.^92^ The improvement of drug solubility and pharmacokinetics resulted in a cytotoxicity profile similar to the free drug.^92^ Docetaxel has also been encapsulated into d‐a‐tocopheryl polyethylene glycol succinate (TPGS)‐based micelles conjugated to cetuximab, a monoclonal antibody specifically binding to EGFR, to actively target EGFR‐expressing TNBC cell lines.^93^ This strategy demonstrated an increased therapeutic effect of targeted docetaxel compared to the free drug.^93^

*Dendrimers*: Dendrimers are synthetic macromolecules of nanometer dimensions (10 nm to 100 nm) formed by repeated units of branched monomers arising radially from a central core.^80^, ^94^ The preparation of dendrimers can be achieved by divergent (from the central core to the periphery) or convergent (from the periphery to the inner core) synthesis.^94^ In both processes controlled polymerization reactions are repeated, adding a precise number of terminal groups at each step or generation.^81^ From the fifth generation onwards, dendrimers present a cavity‐enriched spherical shape, which make them unique vehicles for drug delivery.^95^ Similarly to polymeric micelles, amphiphilic dendrimers with a hydrophobic core and a hydrophilic periphery can be produced. In addition to therapeutic agents, targeting ligands or imaging compounds can easily be conjugated to the functional groups on the dendrimers surface, increasing their functionality.^95^ The ability of dendrimers to deliver gene silencing sequences has also been demonstrated.^96^, ^97^ For instance, four‐generation poly(amidoamine) dendrimers were constructed and conjugated to antisense oligodeoxynucleotides (AODNs) targeting the vascular endothelial growth factor (VEGF). This approach increased the accumulation of dendrimers into a TNBC‐xenograft mouse model and inhibited the expression of VEGF, significantly reducing tumor vascularization compared to the AODNs alone.^96^ Finlay et al. also showed the ability of a modified third generation poly(amidoamine) dendrimer to knockdown the TWIST1 transcriptor factor and its associated target genes in TNBC cells utilizing siRNA sequences. TWIST1 is commonly overexpressed in aggressive breast cancers and is involved in regulation processes of cellular migration through epithelial‐mesenchymal transition (EMT), which makes it a promising target for metastatic TNBC.^97^

*Viral nanoparticles*: Viral nanoparticles are biological systems composed of proteomic and genomic complexes, with protein scaffolds typically packaging viral genetic information.^98^, ^99^ These nanoplatforms naturally occur in a variety of shapes (e.g. icosahedrons, spheres, and tubes) typically ranging from 10 nm to over 1 μm.^98^, ^99^ The natural process of protein scaffold formation by hierarchical self‐assembly of individual protein subunits provides a simple means for drug loading, which constitutes one of the main advantages of viral‐based nanocarriers.^100^ Furthermore, although only a few viruses show a natural affinity for receptors that are upregulated in tumor cells, numerous chemical and genetic engineering techniques allow scientists to design viral particles able to target specific tumors.^80^, ^99^ Various types of virus, including adenovirus and adeno‐associated virus, have been used for this purpose, but bacteriophages (or phages) are one of the most promising ones, due to their lack of tropism for mammalian cells.^101^ Particularly, filamentous bacteriophages have been extremely useful in the screening of homing peptides that target surface proteins of cancer cells via Phage Display for later use as targeting and imaging agents.^102^, ^103^ Moreover, due to their diversity and versatility, these prokaryotic viruses have also been widely used as vehicles for gene and drug delivery.^99^, ^104^, ^105^ Indeed, phages can be simultaneously engineered to display targeting ligands and to carry large payloads of cytotoxic drugs by chemical conjugation, acting as targeted nanomedicines.^104^, ^105^ The potential of filamentous phages displaying a host‐specificity‐conferring ligand and carrying cytotoxic drugs has been demonstrated in breast cancer models. For instance, the use of bacteriophage‐based nanocarriers targeting HER2 overexpressing breast cancer cells showed a greater inhibition of the cell growth compared to the corresponding free drugs.^104^ Although, to our knowledge, no such vehicle has been specifically developed for TNBC therapy, we believe that this class of viral nanoparticles may give an important contribution to the field. On one hand, Phage Display libraries could be used to screen peptides that bind specifically to TNBC cells, thus aiding the identification of novel targets. On the other hand, bacteriophage‐based platforms containing identified binding peptides and loaded with cytotoxic drugs could potentiate the drugs therapeutic effect by selectively destroying cancer cells while reducing side effects. This type of strategy is currently being studied by our research group.

*Liposomes*: Liposomes are spherical vesicles of up to 400 nm in diameter composed of lipids assembled in bilayers surrounding an aqueous core.^79^, ^80^, ^94^ Lipid spheres are spontaneously formed in aqueous environments as amphiphilic molecules favor the contact of their hydrophilic groups with water molecules in an attempt to shield the hydrophobic ones.^94^ Depending on solubility, drugs can be loaded either to the lipid membrane or to the aqueous core.^94^ This feature makes liposomes versatile nanocarriers potentially able to improve drug biodistribution and pharmacokinetics.^79^, ^94^ Additionally, liposomes can behave as passive or active targeting agents, depending on the polymer moieties or targeting agents displayed on their surface.^99^ Numerous studies have shown an enhanced antitumor activity of drug‐carrying liposomes (e.g. doxorubicin, arsenic trioxide, ceramide and sorafenib) in xenograft mice models of TNBC compared to the corresponding free drugs.^106^, ^107^, ^108^ A recent study demonstrated the potential of liposomes to actively target integrin α‐3 overexpressing‐TNBC models when attached to LXY (Cys–Asp–Gly–Phe(3,5‐DiF)–Gly–Hyp–Asn–Cys), a cyclic octapeptide.^109^ This strategy led to the accumulation of co‐administered drugs (doxorubicin and rapamycin) at the tumor site, resulting in an improved antitumor efficacy.^109^ Up to now, a few drug‐loaded liposome formulations, namely EndoTAG‐1 (paclitaxel) and MM‐398 (irinotecan), have reached clinical studies in patients with TNBC.^110^, ^111^

*Carbon nanotubes*: Carbon nanotubes are cylindrical structures formed by benzene rings.^80^ Depending on the number of cylindrical layers they are made of, carbon nanotubes can be classified as single‐walled (one layer) or multi‐walled (multiple layers).^79^ Single‐walled nanotubes typically present 1 nm to 2 nm in diameter and more than 50 nm in length.^79^ The multi‐walled nanotubes' diameter can range from 5 nm to 100 nm.^79^ Naturally, carbon nanotubes are insoluble in any solvent, but they can be chemically modified to become water‐soluble or to incorporate any functional group.^79^, ^80^ The possibility of multiple functionalization of carbon nanotubes to bind a variety of molecules at once, combined with their unique biological and chemical features, makes them an advantageous vehicle for cancer therapy and imaging.^79^, ^80^, ^112^, ^113^ Both the size of the cylindrical structures and the number of walls can affect the mechanism of cellular uptake of nanotubes.^109^, ^110^, ^111^ Usually, single‐walled carbon nanotubes have the ability to penetrate into cells showing localized effects and prolonged distribution, whereas multi‐walled carbon nanotubes are not incorporated by cells.^114^, ^115^, ^116^ An interesting application of multi‐walled carbon nanotubes for TNBC therapy based on photothermal‐induced ablation has been proposed.^117^ By mediating hyperthermia, nanotubes promoted cell membrane permeabilization and necrosis, eradicating both tumor mass and breast cancer stem cells, which are typically resistant to conventional thermal approaches and are involved in tumor recurrence.^117^

*Nanoconjugates*: Nanoconjugates consist of nanoplatforms containing active functional groups covalently bound to therapeutic agents.^99^ Recently, various monoclonal antibody‐ and polymer‐drug conjugates with applications in targeted anticancer therapy have been described.^118^, ^119^, ^120^ Ultra‐small hyaluronic acid‐paclitaxel nanoconjugates were able to target CD44 cancer cell surface receptors and enhance the antitumor efficacy and overall survival over free drug in a mouse model of breast cancer brain metastases.^119^ Moreover, the multifunctionality of poly(*β*‐L‐malic acid) (PLMA) nanoplatfoms was demonstrated by simultaneous conjugation of antitumor nucleosome‐specific monoclonal antibody 2C5, anti‐mouse transferrin receptor (TfR) antibody, and morpholino antisense oligonucleotides (MASONs).^121^ This approach allowed targeting of breast cancer cells, delivering the agents across the endothelial system, and inhibiting EGFR synthesis.^121^ When applied to in vivo models of TNBC, this system was able to significantly inhibit EGFR synthesis and stop tumor progression.^121^ Ljubimova et al. also synthesized PLMA‐based nanoplatforms covalently linked to antisense oligonucleotides targeting EGFR (AON_EGFR_) and α4 (AON_α4_) and β1 (AON_β1_) chains of laminin‐411—the tumor vascular wall protein and angiogenesis marker—combined with TfR monoclonal antibodies for extravasation and targeted tumor uptake.^122^ This strategy showed a synergistic effect of three AONs, compared to their isolated effect, leading to a significant arrest of EGFR and laminin‐411 synthesis and tumor growth without presenting side effects in TNBC xenograft mice.^122^ Recently, EC1456, a folate‐drug conjugate, and IMMU‐132, an antibody‐drug conjugate, entered clinical phase studies in patients with various types of cancer, including TNBC subtype.^123^ EC1456 comprises folate molecules bound to tubulysin B hydrazide, a cytotoxic agent, whereas IMMU‐132 consists of RS7, an anti‐TROP‐2 antibody, conjugated to the active metabolite of irinotecan drug (SN‐38).^123^

#### 2.3.2 Predicting Response to Therapy

Monitoring the patient's response to therapy is a crucial step in a successful treatment to assure a timely alteration of the therapeutic regimen when improvements are not observed. Moreover, and as important as monitoring patients under treatment, is the prediction of their response to therapy even before drug administration.^124^ In theory, predictive biomarkers identifying responsive patients to individual therapies will enable personalized therapeutic approaches, avoiding unnecessary exposure of unresponsive patients and resulting in better outcomes of the responsive subgroups.^125^, ^126^

Over the last years, several gene expression‐based tests that help predicting the clinical outcome and recurrence in breast tumor patients have become commercially available.^127^ For instance, Oncotype DX Breast Cancer Assay and MammaPrint—are used by clinicians to determine the best treatment for patients with early‐stage breast cancer that may have spread to nearby lymph nodes, but not towards distant parts of the body.^128^ Oncotype DX Breast Cancer Assay is a reverse transcription polymerase chain reaction‐based test performed on paraffin embedded tissue sections, whereas MammaPrint, a gene expression microarray analysis, is performed on fresh tissue samples.^128^ However, such a prognosis test for metastatic breast cancer patients including TNBC is still to be developed. In fact, biomarker‐driven therapeutic approaches for TNBC remain an immature field of research.

A couple of factors that may facilitate the development of predictive biomarkers have recently been suggested. On one hand, the assessment of biomarkers for currently available therapeutic agents should focus on features and mechanisms of each individual agent.^125^ On the other hand, biomarkers for novel therapeutic agents must be developed simultaneously from the preclinical phase.^125^ By associating the development of biomarkers to the intrinsic biology of both disease and treatment, shorter periods from research to clinic may be necessary.^125^

Currently, a few biomarker‐driven therapies targeting TNBC have been proposed. For example, a biological therapy with cetuximab and panitumumab monoclonal antibodies targeting EGFR has emerged.^66^, ^128^ High levels of EGFR are commonly found in TNBC and are clinically associated to poor prognosis, which makes EGFR both a potential target and predictive biomarker.^129^, ^130^, ^131^ Mutations in the BRCA1 gene, involved in DNA repair, have also been evaluated as potential response biomarkers of TNBC patients to DNA damaging agents and PARP inhibitors, as BRCA1 alterations are associated with up to 90% of TNBC tumors.^126^ The expression of androgen receptor (AR), although present in less than 35% of TNBC cases, may also represent a potential therapy‐guiding target.^132^, ^133^ Bicalutamide, an AR antagonist, is already in phase II clinical studies.^132^, ^133^ Despite recent efforts, further work is needed to identify reliable and clinically relevant predictive biomarkers, as well as to develop techniques and reference values for their use.

## 3 Challenges and Opportunities

IHC analysis of biopsy samples is so far the best available method for TNBC diagnosis, following the critical examination of patients performed by clinicians. However, when first symptoms are detected, an effective treatment can rarely be provided. Molecular imaging techniques using targeting ligands to increase the specificity of contrast agents represent a promising approach for the early detection of tumors. The success of this strategy is intimately dependent on the utilization of an adequate targeting ligand. Consequently, it is crucial to identify suitable targets expressed in early stages of tumor growth before developing any targeting agents. Despite the diversity of targeting ligands under investigation, the use of their specific receptors as biomarkers of TNBC still needs to be validated in relevant models before claiming their clinical significance. Moreover, some issues, such as the high production costs of antibodies, the variable biodistribution of small peptides and the high susceptibility to degradation of aptamers, must first be overcome before these strategies can be translated to clinical practice. When patients are diagnosed with TNBC, the only available therapeutic options, apart from surgery and radiotherapy, are nonspecifically designed cytotoxic agents, such as anthracyclines and taxanes. Furthermore, the potential resistance to these drugs commonly limits subsequent choices in case the initial treatment does not work adequately. Besides, various clinical trials are focusing on alternative therapeutic agents including drugs, antibodies and inhibitors, either alone or in combination, special attention must be paid to the population subset being studied. To assure a more accurate evaluation of drug‐target interactions, novel trials should be specifically designed for selected populations of TNBC patients carrying the target(s) of interest. The use of drug delivery strategies that actively target cellular receptors expressed by TNBC cells may contribute to improve the efficacy of both conventional and innovative drugs by improving targeting and reducing systemic toxicity and drug resistance. Nanotechnology and synthetic biology have promoted the development of several nanocarriers and targeting agents that enable the delivery of drugs at the desired targets. Despite the potential of targeted nanomedicines, only a few formulations (i.e. polymeric micelles, liposomes and nanoconjugates) have reached clinical stages in the treatment of patients with TNBC. Engineering the appropriate drug delivery system is a complex process. Various relevant factors including biodistribution, pharmacokinetics, biodegradability and immunogenicity must be considered. Additionally, their performance depends on the simultaneous combination of the adequate drug, targeting agent and target population. Currently, the application of these strategies to TNBC populations is greatly limited by the lack of well known, highly expressed targets, as well as by the development of corresponding targeting agents.

From our perspective, the most promising way to solve the TNBC equation would involve a combined approach to all variables, starting with the pathological and molecular characterization of TNBC and tumor microenvironment looking for common patterns, followed by the construction of multifunctional platforms able to image the drug delivery process and monitor the response to therapy. Several techniques that could help researchers in this process are currently available. For instance, Phage Display and SELEX technologies hold great promise to the search for novel targets. On the other hand, nanotechnology‐based carriers offer the necessary versatility to allow the combination of both diagnostic and therapeutic agents in a unique platform.

## 4 Conclusions

The lack of effective and safe therapeutic options for patients diagnosed with TNBC has been driving many research and clinical efforts in this field. Particularly, various drug delivery and targeting agents are under investigation for delivering therapeutic and/or imaging agents at the target tissue. Although nanomedicines with potential application in TNBC are still in early stages of development, this class of medicines has demonstrated capacity to overcome the constraints of current ineffective and cytotoxic therapies. Additionally, nanotechnology‐based targeting ligands, such as peptides, aptamers and small molecules promise to contribute to the improvement of detection technologies, which are commonly a limiting step for a timely therapeutic intervention in this group of patients. Although further endeavors are still needed to validate the clinical relevance and feasibility of these nano‐based strategies, novel nanocarriers, either man‐made (synthesized) or of natural origin, should be equipped with ligands that specifically target TNBC‐receptors and, preferably, conjugated with more than one anticarcinogenic compound to reduce occurrence of resistance and increase the success rate of the treatment.
